# Liesegang rings in the setting of end‐stage renal disease

**DOI:** 10.1002/iju5.12494

**Published:** 2022-06-29

**Authors:** Alexander R Gross, Shahrier Amin, Tomislav Jelic

**Affiliations:** ^1^ Department of Pathology Anatomy, and Laboratory Medicine WVU Robert C Byrd Health Sciences Center Morgantown West Virginia USA; ^2^ Department of Pathology Charleston Area Medical Center Charleston West Virginia USA

**Keywords:** diabetic proteinuria, kidney stones, liesegang rings

## Abstract

**Introduction:**

Liesegang rings are acellular, lamellar, concentric rings of organic or inorganic material naturally formed in both biologic and environmental systems. Description in human tissue is scarce. Liesegang rings have exclusively been identified in association with pathologic disease processes and thus are not typically considered in differential diagnosis. They are usually described with cystic or inflammatory lesions. Histologically, Liesegang rings show features that are also seen in sections of parasitic ova, larvae, psammoma bodies, and by radiology as calcifications in cystic diseases of the breast and kidney.

**Case presentation:**

We noted at autopsy of a 59‐year‐old diabetic woman multiple black “stones” in the renal medulla. Microscopic examination demonstrated these to contain Liesegang rings.

**Conclusion:**

Liesegang rings formation should be considered in the differential diagnosis of atypical appearing deposits in the kidneys and other tissues. They may play a role in the pathogenesis of kidney stones.

**Abbreviations & Acronyms**
CT: computerized tomography
ESRD: end‐stage renal disease
LR: Liesegang ring
PAS: Periodic acid‐Schiff

**Keynote message**

Liesegang rings – target‐like concentric precipitations of organic or inorganic material – can mimic kidney stones. Liesegang rings can originate from precipitation of glycoproteins in the kidney medulla of patients with diabetic proteinuria.

## Introduction

Occasionally observed in clinical medicine, “Liesegang ring (LR)” refers to a chemical precipitation pattern first described by F. F. Runge in 1855 based on filter paper chromatography work he performed with textile dyes.^1^ R.E. Liesegang then demonstrated the phenomenon empirically in 1896 by inorganic thin layer chemistry; precipitate of silver nitrate formed an alternating series of dark and clear radial striations on a layer of potassium dichromate gel.^2^ LRs form natural structures within sedimentary rock. The LR phenomenon is also present at hydrothermal seafloor seepages in mollusk shells. It is additionally utilized industrially via electrodeposition in silicon chips. A precipitate film usually forms over a pre‐existing nidus/bubble surface. With growth, the film fractures into polygonal sections that can move by fluid flow yielding more space and opportunity for further growth. Material at the contact line (at which tube, bubble, and solution meet) deposits rings in a concentric fashion.^3^ Their formation is also noted within the human body.

Whether in humans or nature, the LR pattern of periodic precipitation is described chemically and mathematically.^4^, ^5^ The medical significance of LRs is unclear though and is primarily cited as an incidental finding or diagnostic pitfall. Cystic or inflammatory processes may potentially play a role in their formation. Due to the characteristic concentric ring morphology seen on histologic sections LRs can be mistaken for parasitic ova.^6^ Body sites identified with LRs include the paranasal sinus, breast, eye, genitourinary system, and retroperitoneum.^7^, ^8^, ^9^, ^10^

In vivo LRs primarily form in colloidal systems that are supersaturated. A mechanism of competing effects of diffusion, precipitation, and supersaturation is theorized. Concentric rings deposited are spaced according to a mathematical law in a spherical distribution around an amorphous core. The ring width begins on the order of nanometers and increases with the ring number from the core; the space between rings increases with the time taken to form them.^5^ The laminations are typically birefringent and can be characterized with histochemical stains: Periodic‐acid Schiff, Diff‐Quick, Masson's trichrome, Papanicolaou, Gram stain, hematoxylin–eosin, and von Kossa.^11^, ^12^, ^13^, ^14^, ^15^

## Case presentation

A 59‐year‐old‐woman with a history of insulin‐dependent diabetes mellitus, hypertension, and end‐stage renal disease was admitted to the hospital with intermittent chest tightness, shortness of breath, and multiple episodes of epistaxis. Profuse bleeding was noted from an arteriovenous dialysis graft. Her recent surgical history was significant for mitral valve replacement for which she was taking Coumadin. Recent laboratory values revealed 100 mg/dL urine protein, few amorphous crystals, and urine pH of 6.0. On admission, her diagnostic testing showed INR 8, glucose 322, and chest CT showing large pleural effusion. Her hospital course was complicated by hypotension refractory to maximal intensive care and massive transfusions. She expired and an unrestricted autopsy was requested.

**Fig. 1:**
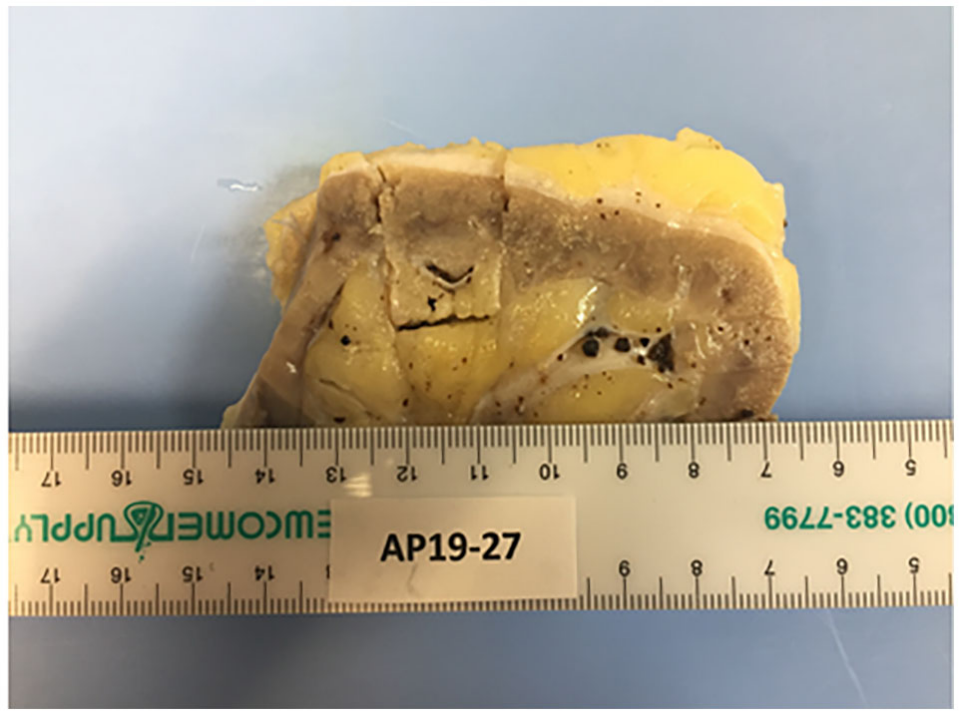
A section of kidney at autopsy. Multiple small black concretions are shown within the renal pelvocalyceal system consistent with renal calculi or Liesegang rings.

**Fig. 2:**
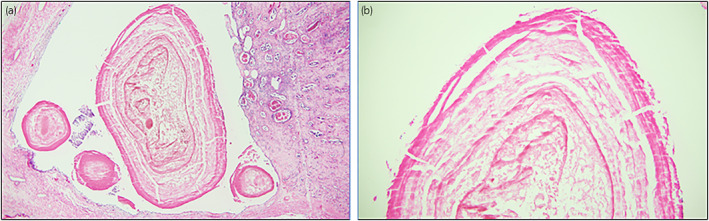
(a) Histologic sections of kidney to include renal pelvocalyceal system showing concentric eosinophilic lamellae distributed around an amorphous core in the calyx. H&E, 40×. (b) Lamellae show varying widths and intra‐ring interval consistent with periodic Liesegang ring formation. H&E, 100×.

**Fig. 3:**
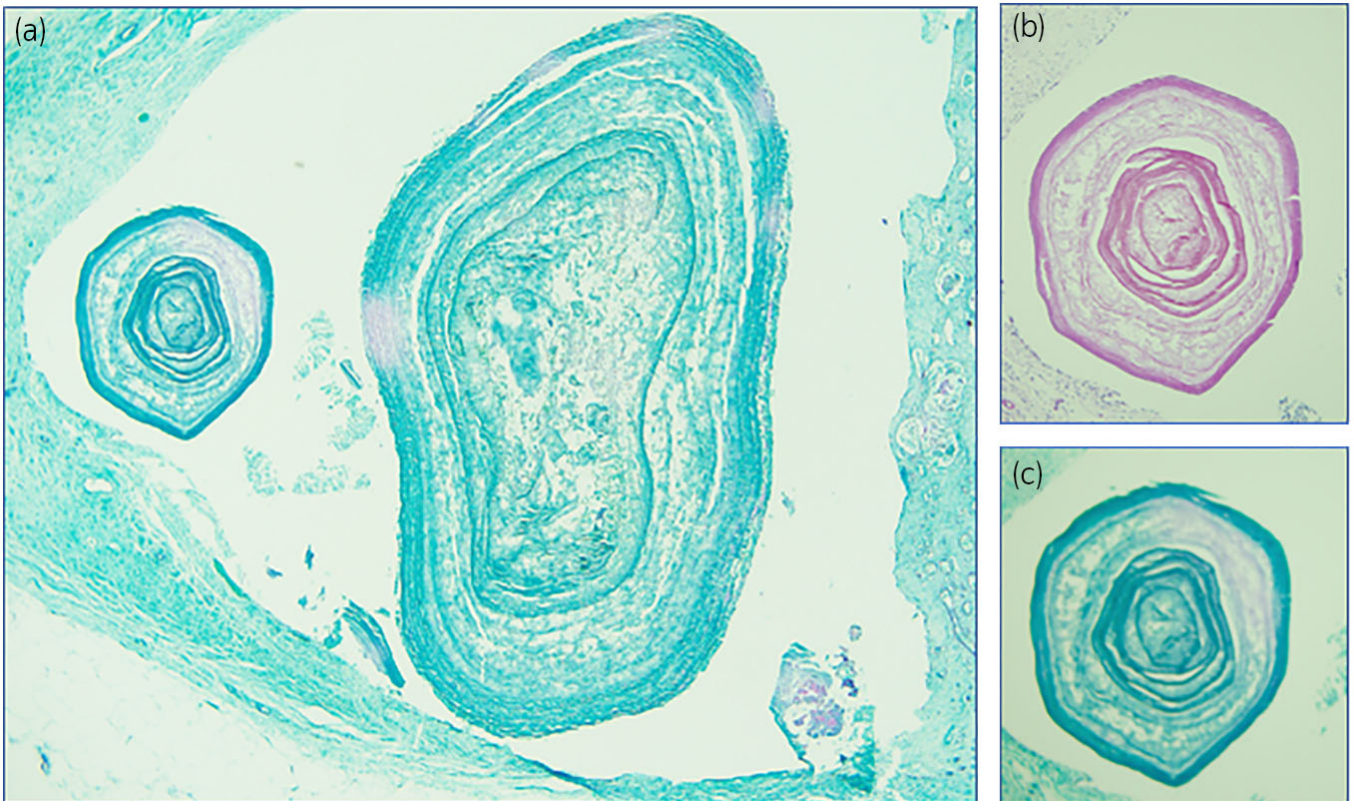
(a) Highlighted concentric lamellae varying in thickness and distance. PAS light green, 40×. (b) Characteristic Liesegang ring structure. PAS, 100×. (c) Characteristic Liesegang ring structure. PAS light green, 100×.

Post‐mortem examination confirmed that the cause of death was massive bleeding (predominantly right thoracic cavity) because of Coumadin overdose. Contributory factors were renal insufficiency, cardiomegaly, and possible sepsis. Numerous small black concretions ranging from 0.1–0.3 cm were present in the pelvocalyces of both kidneys; ureters were unremarkable (Fig. 1). Microscopic examination of the concretions demonstrated globules of amorphous material encircled by concentric layers of birefringent, collagenous fibers consistent with LRs (Figs. 2,3). Small PAS positive globules reminiscent of Tamm‐Horsfall protein casts were noted in the atrophic tubules and medulla. Renal histology also revealed nodular Kimmelstiel‐Wilson structures with advanced global glomerulosclerosis, Mönckeberg calcific medial sclerosis, severe arteriosclerosis, and severe interstitial fibrosis and tubular atrophy. There were no features suspicious for renal disease of the immune complex type or paraprotein‐related disease.

## Discussion

We discovered LRs incidentally on post‐mortem examination. However, Liesegang rings in biopsies can also represent an important diagnostic pitfall which may lead to diagnostic odyssey and treatment delay. More study of their pathogenesis is needed. Table 1 shows a variety of conditions where LR‐like concretions can be seen. Morphologically in hematoxylin–eosin stained tissue, LRs can mimic parasitic ova which display a refractile shell and amorphous ovum at the core. Radiologically their features may suggest a neoplastic or inflammatory process. These may also co‐exist with LRs in the same anatomical site.^16^ LR‐like concretions can also be seen in malakoplakia,^17^ benign cysts,^14^ inflammation,^9^ or neoplasms.^13^ Additional renal LR associations are cysts/cyst fluid^18^, ^19^, ^20^ and hematuria.^21^

**Table 1:** Properties of Liesegang Rings and their common mimics

Pathologic Entity	Histomorphology (Hematoxylin and Eosin)	Histochemical Reactivity/*Physical Properties*	Clinicopathologic Significance
**Liesegang ring**	Extracellular eosinophilic globular core surrounded by concentric rings of variable thickness, some with fine radial striations (7–800 μm)	Von Kossa (+), PAS (+), Gram (+)/*Collagen, Fe, Si, S, Ca*	Undetermined
Michaelis‐Guttman bodies	Intracytoplasmic, basophilic concentric rings of decreasing thickness in macrophage (5–15 μm)	Von Kossa (+), PAS (+), Gram (−)/*Fe, Ca*	Malakoplakia
Parasite ova	Extracellular eosinophilic amorphous core with laminated outer ring (shell), ovoid to barrel shaped (60–84 μm)	Von Kossa (−)	*A. lumbricoides, D. renale*
Corpora amylacea	Intraluminal/interstitial, eosinophilic/ amphophilic/basophilic, concentrically lamellated spherical to angulated bodies (2–20 μm)	Von Kossa (−), PAS (−)/*Protein*	Physiologic brain, kidney, prostate
Collagen spherules (breast)	Intraluminal clusters of eosinophilic spherules of basement membrane material, some with radial fibrils or microcalcification (20–100 μm)	Von Kossa (+), PAS (+)/*Basement membrane‐like material*	Collagenous spherulosis
Tamm‐Horsfall protein (hyaline or uromodulin)	Renal tubule‐contoured, intraluminal homogenous to frothy eosinophilic smooth‐irregular edges (20–200 μm)	Hyaline: PAS (+), homogenous/*Uromodulin + unknown material* Uromodulin: PAS (+), “bubbly”/*Pure uromodulin*	Urinary tract infection, cast nephropathy, tubulointerstitial nephritis, urolithiasis, physiologic
Light chain casts	Renal tubule‐contoured, intraluminal homogenous pale eosinophilic material, contoured edges (20–200 μm)	PAS (+,“glassy”), Uromodulin (−), Kappa or lambda (+)/*Polypeptide*	Plasma cell dyscrasia (multiple myeloma, plasma cell myeloma, gammopathy)
Renal calculi	Single/multiple eosinophilic spherules/polygons, some with concentric rings, homogenous to frothy, intraluminal or interstitial (10 – 1 × 10^9^ μm)	Commonly Von Kossa (+), Commonly PAS (−), Commonly Masson trichrome (−)/*Ca, Uric acid, PO4, Oxalate, Cysteine, Xanthine*	Urolithiasis, urinary tract infection
Psammomatous calcification	Round basophilic calcifications with concentric laminations (20–200 μm)	Von Kossa (+), PAS (−), Masson trichrome (−)/*Ca, P, Mg, Na, Fe and Zn*	Papillary thyroid/renal cell carcinoma, ovarian cystadenoma, meningioma

LR formation may play a role in the formation of renal calculi. LRs admixed with calcium oxaloacetate crystals were described in urine cytology.^22^ LRs may be under‐reported since grossly LRs and renal calculi are challenging to distinguish. LR phenomenon may also have a role in the formation of renal stones; calculi are often not examined microscopically thus the exact correlation is difficult to ascertain. We describe LRs arising in the setting of severe end‐stage diabetic kidney disease and dialysis. Possible sources of a nidus in the kidney include Tamm‐Horsfall casts, infections, thrombi, pre‐existing calculi, etc. We hypothesize that concentric LRs form by apposition of PAS positive glycoproteins in pelvocalyces by incorporation of collagen fibers. Prolonged glucosuria/proteinuria, electrolyte abnormalities, and renal concentration of medications could potentially favor the formation of LRs. This maybe further influenced by changes to urine output. Thorough analysis of serum/urine content, medication history, and renal filtration/output should be pursued in cases where suspicion is high for LRs, or if they are already diagnosed.

The atomic structure and composition of LRs in humans is not well described. Radiography, electron microscopy, and special staining all have revealed traces of iron, silica, and sulfur. Some have noted calcium among various ionic constituents. However, larger studies with more precise chemical analysis are needed. Also important is the continued investigation upon the mechanistic variables of pH, temperature, concentration, time and medium.

We present here an example of LRs mimicking renal calculi associated with end‐stage renal disease due to diabetic nephropathy. Although a clear association of LRs to human pathology is documented no mechanism yet explains their various clinical presentations. Our case suggests that ESRD and dialysis may constitute a possible medium for LR formation. Case reports such as this are needed to promote awareness of LR and thus assist in the formulation of appropriate treatment plans, especially when neoplastic or infectious diseases are within the differential diagnosis. Furthermore, additional case report may serve as the basis for directed scientific experiments.

## Conflict of interest

The authors declare no conflict of interest.

## Approval of the research protocol by an Institutional Reviewer Board

Not applicable.

## Informed consent

Not applicable.

## Registry and the Registration No. of the study/trial

Not applicable.

## Author Contributions

Shahrier Amin: Conceptualization; formal analysis; supervision; writing – review and editing. Tomislav Jelic: Conceptualization; data curation; formal analysis; project administration; writing – review and editing.
